# New Roles for Corticosteroid Binding Globulin and Opposite Expression Profiles in Lung and Liver

**DOI:** 10.1371/journal.pone.0146497

**Published:** 2016-01-07

**Authors:** Jose Gulfo, Angelo Ledda, Sabrina Gea-Sorlí, Laia Bonjoch, Daniel Closa, Mar Grasa, Montserrat Esteve

**Affiliations:** 1 Department of Nutrition and Food Sciences, Faculty of Biology, University of Barcelona, Barcelona, Spain; 2 CIBER Obesity and Nutrition, Institute of Health Carlos III, Madrid, Spain; 3 Department of Experimental Pathology, IIBB-CSIC-IDIBAPS, Barcelona, Spain; University of Szeged, HUNGARY

## Abstract

Corticosteroid-binding globulin (CBG) is the specific plasma transport glycoprotein for glucocorticoids. Circulating CBG is mainly synthesized in liver but, its synthesis has been located also in other organs as placenta, kidney and adipose tissue with unknown role. Using an experimental model of acute pancreatitis in *cbg*^*-/-*^ mice we investigated whether changes in CBG affect the progression of the disease as well as the metabolism of glucocorticoids in the lung. Lack of CBG does not modify the progression of inflammation associated to pancreatitis but resulted in the loss of gender differences in corticosterone serum levels. In the lung, CBG expression and protein level were detected, and it is noteworthy that these showed a sexual dimorphism opposite to the liver, i.e. with higher levels in males. Reduced expression of 11β-HSD2, the enzyme involved in the deactivation of corticosterone, was also observed. Our results indicate that, in addition to glucocorticoids transporter, CBG is involved in the gender differences observed in corticosteroids circulating levels and plays a role in the local regulation of corticosteroids availability in organs like lung.

## Introduction

Acute pancreatitis is a serious inflammatory process with significant morbidity and mortality. The most relevant complication during acute pancreatitis is the systemic inflammation that, in the severe forms of the disease, may lead to an acute respiratory distress syndrome [1]. This pulmonary dysfunction is characterized by an influx of inflammatory leukocytes and increases in pulmonary vascular permeability, being one of the most important factors contributing to death during the first week of the disease [2]. The mechanisms responsible for the involvement of distant organs are still unclear and different pathways have been suggested, including oxygen-derived free radicals and cytokines [3].

As occurs with other critical illnesses, such as sepsis, trauma and septic shock, the hypothalamic–pituitary–adrenal axis plays an important modulatory role in the control of the inflammatory process. Several reports suggested a link between an impaired adrenal secretion and the progression of systemic inflammation in acute pancreatitis [4]. In addition, corticosteroid insufficiency has been reported in patients with acute pancreatitis [5]. Nevertheless, the use of corticosteroid in the treatment of acute pancreatitis is still being debated and experimental studies suggest that although the prophylactic use of corticosteroids showed efficacy on some features of the disease, this effect was not observed with the therapeutic use [6].

In addition to glucocorticoids, changes in the levels of corticosteroid-binding globulin (CBG) have also been reported in patients with acute pancreatitis [7][8]. CBG is the specific high-affinity plasma transport glycoprotein for glucocorticoids. It is mainly synthesized in liver, although it could be also produced by the placenta, kidney and adipose tissue [9][10][11]. The main function of CBG seems to be glucocorticoids transport since under normal conditions 80–90% of circulating cortisol is bound with high affinity to CBG, while only 10–15% binds with low affinity to albumin and the remaining 5–10% is known as “free cortisol” [12].

It is accepted that CBG actively deliver glucocorticoid to inflamed tissue due to the action of elastase released by activated neutrophils. This protease cleaves CBG and disrupts the glucocorticoid-binding site, thus resulting in the release of the corresponding glucocorticoid in the areas of inflammation [13]. However, CBG has been located also in some intracellular compartments suggesting additional, and yet unknown, functions [14].

Herein, our study was designed to investigate, in an experimental model of acute pancreatitis in mice, whether changes in CBG could affect the progression of the disease as well as the metabolism of glucocorticoids in the lung. To do this, we compared the effects of pancreatitis in *cbg*^*+/+*^ and in *cbg*^*-/-*^ animals. Moreover, considering the remarkable differences in the metabolism of glucocorticoids between males and females, we did the experiments comparing the effects between the two genders.

## Materials and Methods

Male and female C57BL/6 mice (25–30 g b.w.) *cbg*^+/+^ and *cbg*^-/-^ were used in all experiments. The colony of *cbg*^-/-^ was established through crossing heterozygous breeding kindly provided by Dr. Willnow. The procedure followed for disruption of the CBG gene was described by Petersen et al. [15]. Animals were housed in a controlled environment, fed with standard laboratory pelleted formula (Teklad Global 2018, Harlan-Interfauna Ibérica, Sant Feliu de Codines, Spain) and tap water ad libitum. This study conformed to European Community for the use of experimental animals and the institutional committee of animal care and research (C.E.E.A. Universitat de Barcelona) approved it.

### Animal model of acute pancreatitis

Mice received 10 hourly intraperitoneal injections of 50 μg/kg cerulein (Sigma, USA) or PBS as control and were sacrificed one hour after the last injection [16]. Samples of pancreas, liver and lung were obtained, immediately frozen and maintained at -80°C until processed. Lung samples were also obtained for histological study. Samples of blood were centrifuged to obtain serum. Taking into account that some parameters evaluated show important circadiary changes, the experiments were performed at the same hour, being the procedure started at 8 a.m. and animals sacrificed at 7 p.m.

### Lipase

Plasma lipase was determined by using commercial turbidimetric assay kits from Randox (Antrim, U.K.), according to the supplier's specifications.

### Myeloperoxidase

Neutrophilic infiltration was assessed by measuring myeloperoxidase (MPO) activity. MPO was measured photometrically with 3,3',5,5'-tetramethylbenzidine as a substrate. Tissue samples were homogenized with 0.5% hexadecyltrimethylammonium bromide in 50 mM phosphate buffer at pH 6.0. Homogenates were disrupted for 30 seconds using a Labsonic sonicator (Braun Biotech, Inc., Allentown, PA, USA) at 20% power and submitted to three cycles of snap freezing in dry ice and thawing before a final 30 second sonication. Samples were incubated at 60°C for 2 hours and then spun down at 4000 xg for 12 minutes. The supernatants were collected for MPO assay. Enzyme activity was assessed photometrically at 630 nm.

### RNA isolation and RT-PCR

Total RNA from tissue samples were extracted using the TRI Reagent^®^ Solution (Ambion, Inc, USA). The RNA was quantified by measurement of the absorbance at 260 and 280 nm using a NanoDrop ND-1000 spectrophotometer (NanoDrop Technologies, USA).

cDNA was synthesized using the an MMLV reverse transcriptase (Promega, USA) and oligo-dT primers, reverse transcription was then performed from 2 μg RNA sample. The reaction was incubated at 72°C for 5 min and 42°C 60 min, and then stored at -80°C.

Subsequent PCR amplification was performed in ABI PRISM 7900 HT detection system (Applied Biosystems), and was carried out using 10 μl of amplification mixtures containing SYBR Green PCR Master Mix (Life Technologies), 8 ng of reverse-transcribed RNA and 300 nM of the correspondent mice primers: CBG forward: 5’-CCACCAAAGACACTCCCTTG-3’ reverse: 5’-GCACATTCCCTTCATCCAGT-3’; 11β-HSD1 forward: 5’-CAAGGTCAACGTGTCCATCA-3’ reverse: 5’-TCCCAGAGATTTCCTTCATAGC-3’; 11β-HSD2 forward: 5’-CTCCAAGGCAGCAATAGCAC-3’ reverse: 5’-CGTTTCTCCCAGAGGTTCAC-3’; and RPL32 forward: 5’-ACCAGTCAGACCGATATGTGAAAA-3’ reverse: 5’-TGTTGTCAATGCCTCTGGGTTT-3’. Reactions were performed in duplicate and threshold cycle values were normalized to RPL32 gene expression. The specificity of the products was determined by melting curve analysis. The ratio of the relative expression of target genes to RPL32 was calculated by using the ΔC(t) formula.

### Histological study

For histological studies, tissue samples were fixed in 10% neutral buffered formalin, paraplast-embedded, cut into 5 μm thick sections and stained with hematoxylin–eosin according to standard procedures. Sections were evaluated by light microscopy and the severity score was calculated based in a semiquantitative evaluation scale [17].

### Immunohistochemistry

Briefly, the sections were deparaffinized, rehydrated and washed in PBS-Tween, and then they were treated with 0.3% hydrogen peroxide, blocked with 25% Rabbit serum and incubated overnight at 4°C with goat-antimouse CBG (LS-C39044, LifeSpan BioSciences, USA) diluted 1:100. After that, sections were sequentially incubated with biotinylated rabbit anti-goat (1:400, Vector Labs, USA) and avidin–biotin complex reaction (1:200, ABC Elite Kit, Vector Laboratories) and developed with a diaminobenzidine hydrochloride chromogen (Sigma, USA). The slides were scanned and visualized using the Pannoramic Viewer 1.15.2 software.

### Western blot of CBG

Serum samples (100μg per well) were separated by SDS/PAGE in a 10% gel and electrotransferred on to a PVDF membrane (Millipore, Billerica, MA, U.S.A.). After blocking nonspecific binding sites with 5% nonfat milk and in TBSTween 0.05% for 90 minutes at room temperature, the membranes were incubated overnight with a CBG goat anti-mouse polyclonal antibody (1:1,000; LifeSpan) and albumin as control (1:50.000; sc-46293 Santa Cruz Biothecnolgy, USA)

Protein fraction from samples of lung was isolated using the TRI Reagent^®^ Solution (Ambion, Inc, USA). Protein concentration was quantified by QuantiPro^™^ BCA Assay Kit (Sigma, St. Louis, MO, USA) and 30 μg of total protein per well were separated by SDS/PAGE proceeding similarly to serum samples for CBG detection. For lung the control was made using a primary antibody against tubulin (dil 1:1000, DM1A, Abcam, U.K.).

Immunoreactive proteins were further detected by an anti-goat horseradish peroxidase-conjugated secondary antibody (1:20,000; sc-2922, Santa Cruz Biotechnology, USA) and using the Luminata^™^ Forte Western HRP Substrate (Millipore, Billerica, USA).

### Total Corticosterone

Corticosterone in serum was measured by radioimmunoanalysis using a sheep anti-corticosterone antibody (AB1297, Millipore, USA) and we proceeded as described previously [18].

### CBG binding capacity

To remove corticosterone from serum samples, 10 μl of serum samples were added to 0.5 ml of a charcoal-dextran suspension [0.05% dextran and 0.5% charcoal (Norit A, Sigma) in PBS with 0.1% of gelatin (PBSG) at pH 7.4] and incubated at room temperature for 30 minutes with occasional shaking. Samples were then centrifuged and the clear supernatants were further diluted 1/5 with PBSG buffer. These hormone-stripped samples were used for corticosterone binding assays. The final concentration of labelled corticosterone in the tubes was 15 nM in a total volume of 200μl. Then, 200 μl of a constant stirring charcoal-dextran suspension were added to each tube. These were shaken, left to stand for 10 min in an ice bath, and centrifuged for 10 min. Aliquots of 200 μl of the clear supernatants were added to 4 ml of a water-miscible scintillation cocktail (Ecolite, MP Biomedicals, USA) and counted in a standard scintillation counter. In all samples, individual unspecific binding (measured in a 500-fold excess of non-radioactive corticosterone) was measured and subtracted from the total radioactivity bound to diluted stripped serum.

### Statistical analysis

The data were analyzed using the GraphPad software program version 5.0 and were expressed as the mean ± SEM. Statistical comparisons were made by two-way ANOVA analysis, evaluating the variables "treatment" (control, cerulein) and "sex" (male, female). Bonferroni was used as a post-test. The P values < 0.05 were considered significant.

## Results

### Induction of acute pancreatitis

**Fig 1:**
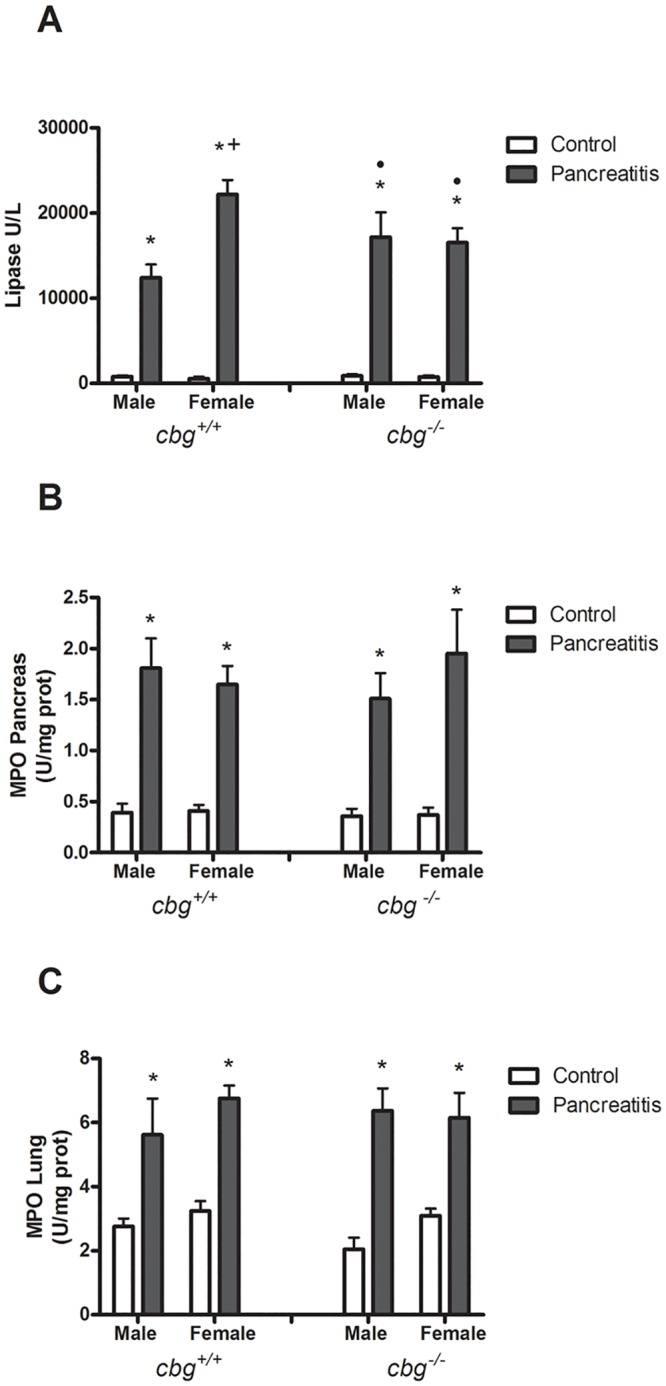
Induction of pancreatitis. (A) Levels of circulating lipase. Pancreatitis resulted in an increase in plasma lipase in all experimental groups. This increase was significantly higher in female *cbg*^*+/+*^. (B) Mieloperoxidase activity in pancreas and (C) lung. Pancreatitis resulted in an increase in myeloperoxidase in all experimental groups. This increase resulted more marked in pancreas than in lung. Two Way Anova: circulating Lipase: Treatment P<0.0001, Sex P = 0.0063 Interaction P = 0.0040; Mieloperoxidase activity in pancreas: Treatment P<0.0001, Sex P = 0.7436, Interaction P = 0.7499; Mieloperoxidase activity in lung: Treatment P<0.0001, Sex P = 0.5030, Interaction P = 0.6427; Bonferroni Post-test: * P<0.05 control vs pancreatitis; + P<0.05 male vs female; • P<0.05 *cbg*^*+/+*^ vs *cbg*^*-/-*^.

Administration of cerulein induced an acute pancreatitis evidenced by the increased levels of circulating lipase. In the *cbg*^*+/+*^ mice, this increase was higher in females than in males. By contrast, there no gender differences were observed in the *cbg*^*−/−*^ mice. In males the increase of lipase circulating levels promoted by pancreatitis was higher in *cbg*^*−/−*^ than *cbg*^*+/+*^ mice, but in female the inverse effect was observed. (Fig 1A).

**Fig 2:**
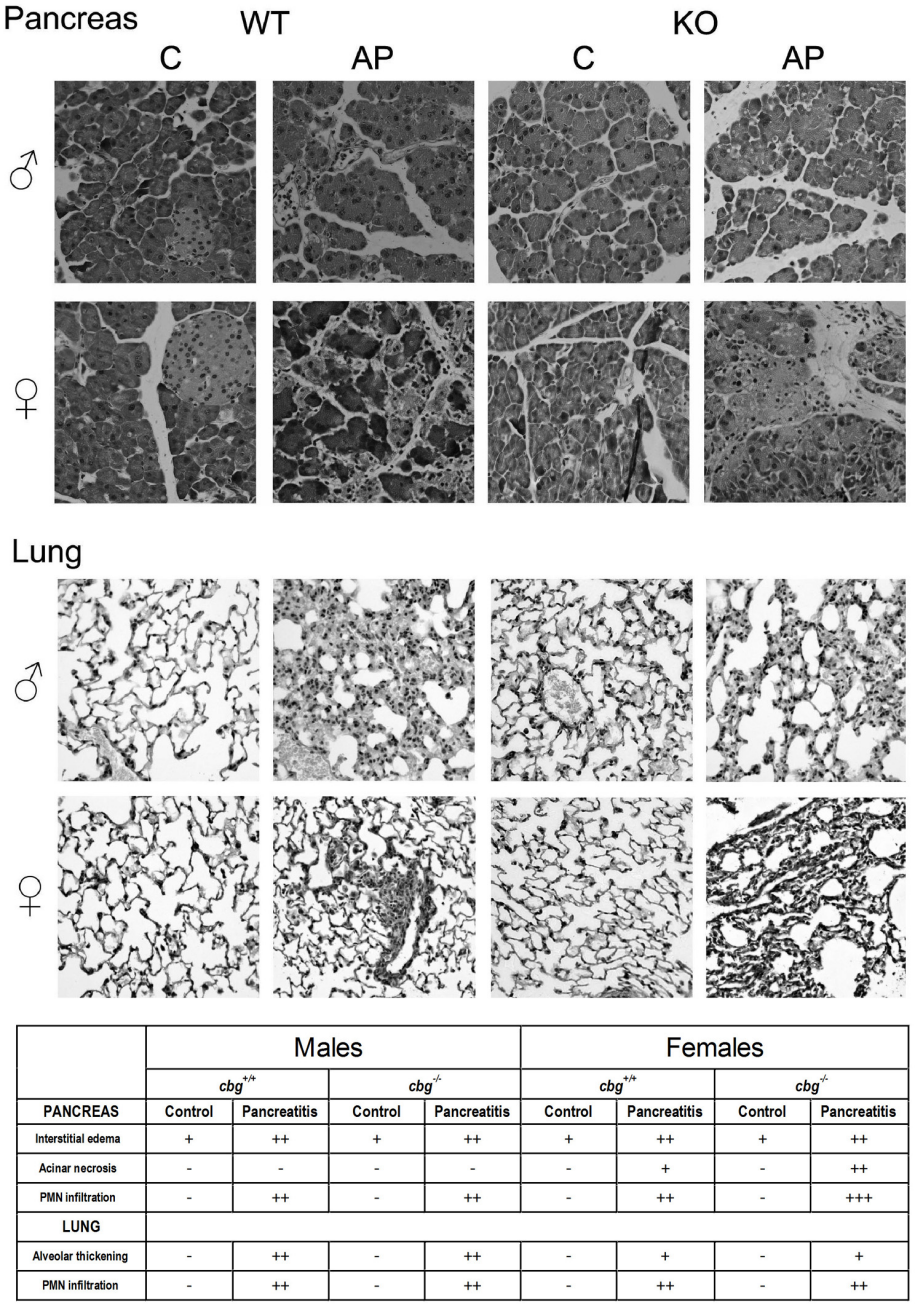
Injury and severity scores for pancreas and lung. Representatives H&E stained sections and severity score for each experimental group. Severity score was obtained using semiquantitative evaluation scale and the results were the mean of the lesions observed in each group. PMN = polymorphonuclear leukocytes;— = no lesion; + = mild lesion; ++ = moderate lesion; +++ = intense lesion.

Inflammation in the pancreas, measured as MPO activity, showed an increase between four to five times in all cerulein treated mice compared to controls without differences among males and females. (Fig 1B). Similar profile was observed in the lung (Fig 1C) but in this case the increase observed in pancreatitis induced mice was between two and three times higher than controls. Histological study (Fig 2) of pancreas showed interstitial edema and infiltration of polymorphonuclear leukocytes (PMN) in all cerulein treated groups. Acinar necrosis was only promoted by cerulein in females. In lung cerulein induced alveolar thickening and polymorphonuclear leukocytes infiltration in all treated mice.

### Corticosterone and CBG in serum and liver

**Fig 3:**
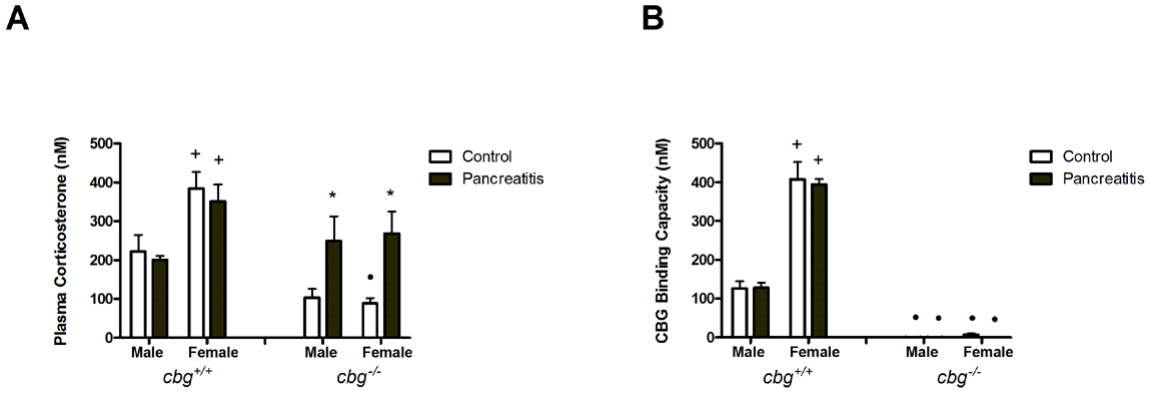
(A) Total corticosterone circulating in serum and (B) CBG binding capacity in serum. Female mice showed higher levels of corticosterone and CBG binding capacity than males in *cbg*^*+/+*^ animals. These differences disappeared in *cbg*^*-/-*^ animals. *cbg*^*-/-*^ females showed significantly lower levels of corticosterone. Pancreatitis did not modify the levels of corticosterone in *cbg*^*+/+*^ animals, while in *cbg*^*-/-*^ mice induced a recovery to levels of *cbg*^*+/+*^ animals. Two Way Anova: Total corticosterone: Treatment P = 0.0210, Sex P<0.0001, Interaction P = 0.0171; CBG binding capacity: Treatment P = 0.7538, Sex P<0.0001, Interaction P = 0.9624; Bonferroni Post-test: * P<0.05 control vs pancreatitis; + P<0.05 male vs female; • P<0.05 *cbg*^*+/+*^ vs *cbg*^*-/-*^.

Pancreatitis did not induce significant changes in *cbg*^*+/+*^ animals, although females showed higher levels of circulating corticosterone than males. Interestingly, the *cbg*^*−/−*^ mice showed lower levels of corticosterone in control animals and cerulein treatment induced recovery of the levels to the *cbg*^*+/+*^ in both males and females (Fig 3A).

Similar results were found when measuring the CBG binding capacity in serum of *cbg*^*+/+*^ animals; females showed higher levels than males and, in both genders, pancreatitis did not modify these levels. As expected, all *cbg*^*−/−*^ mice showed negligible binding activity (Fig 3B).

**Fig 4:**
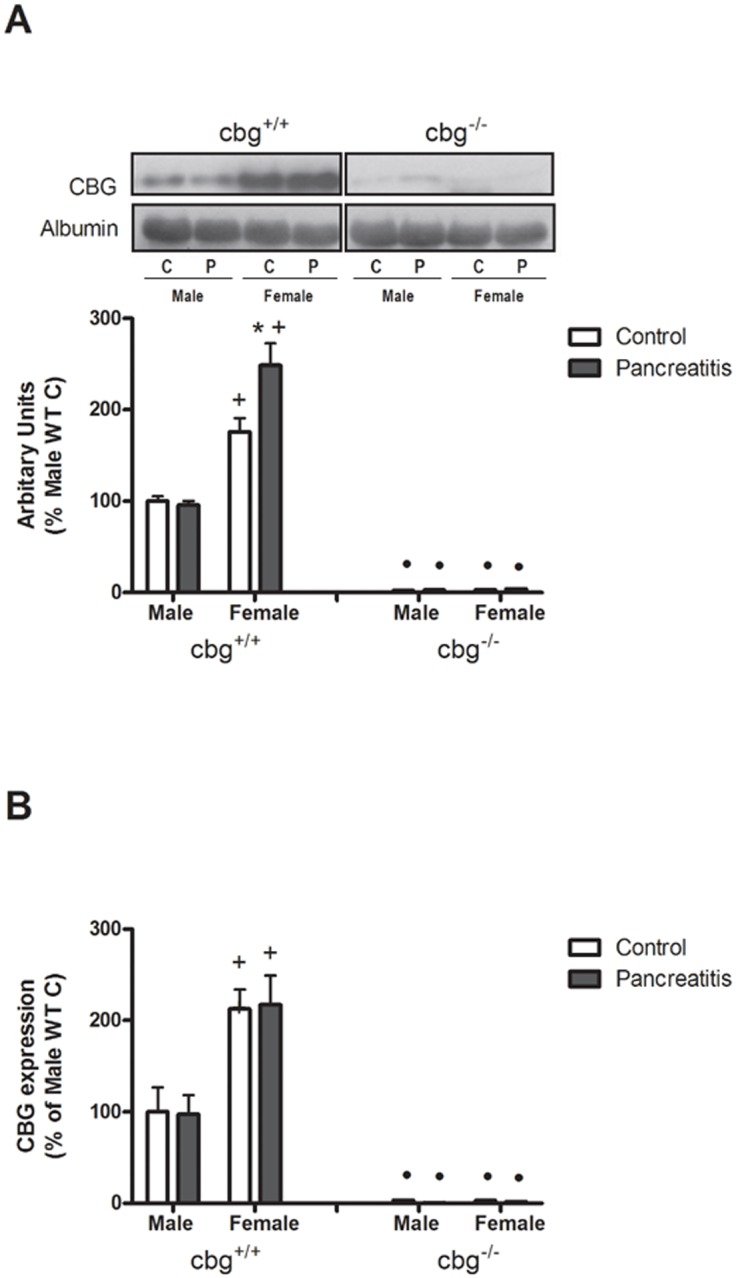
(A) Serum CBG protein levels measured by western blot and (B) liver CBG expression measured by RT-PCR. In both cases, females had higher levels than males and pancreatitis promoted an increase of CBG protein levels only in females. No CBG was detected in serum of *cbg*^*-/-*^ mice and only a residual expression was detected in liver. C refers to control mice and P refers to pancreatitis mice. Two Way Anova: Serum CBG levels: Treatment P = 0.0155, Sex P<0.0001, Interaction P = 0.0007; liver CBG expression: Treatment P = 0.9681, Sex P<0.0001, Interaction P = 0.9972; Bonferroni Post-test: * P<0.05 control vs pancreatitis; + P<0.05 male vs female; • P<0.05 *cbg*^*+/+*^ vs *cbg*^*-/-*^.

Circulating CBG levels in *cbg*^*+/+*^ animals revealed that females had higher levels than males. Pancreatitis did not induce changes in males but resulted in an increase in CBG in females. No detectable levels of CBG were found in *cbg*^*-/-*^ animals (Fig 4A).

Finally, liver is the main source of circulating CBG and, as occurred with the serum levels, significant differences were found when comparing males and females in *cbg*^*+/+*^ animals (Fig 4B). The higher levels of expression were observed in females and pancreatitis had no effect on this expression. A residual expression of CBG was detected in *cbg*^*-/-*^ mice.

### CBG and corticosterone metabolism in the lung

**Fig 5:**
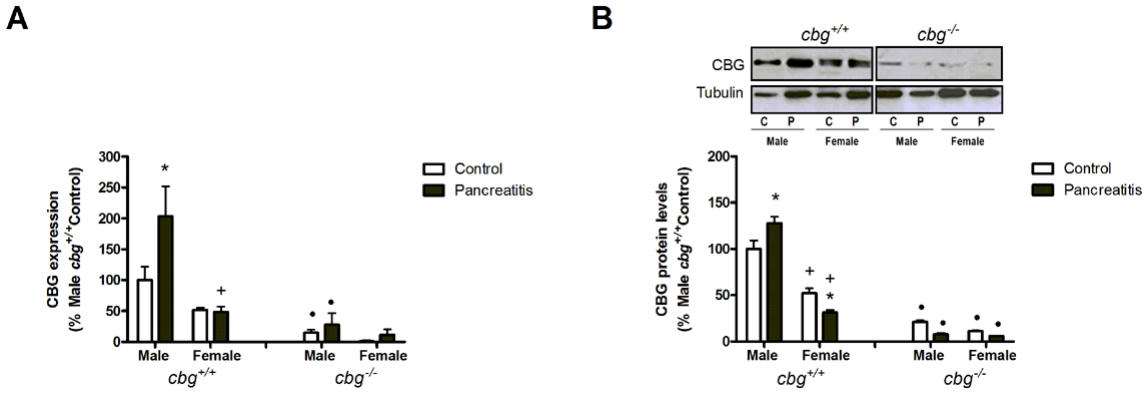
(A) Lung CBG expression measured by RT-PCR and (B) lung CBG protein levels measured by western blot. Females had significantly lower levels of CBG protein than males without differences in the expression levels. Only in mice males the both parameters were increased by induction of pancreatitis. A residual expression and levels of CGB were detected in *cbg*^*-/-*^ mice. C refers to control mice and P refers to pancreatitis mice. Two way Anova: Lung CBG expression: Treatment P = 0.0182, Sex P<0.0001, Interaction P = 0.0188; lung CBG levels: Treatment P = 0.4544, Sex P<0.0001, Interaction P = 0.0009; Bonferroni Post test: * P<0.05 control vs pancreatitis; + P<0.05 male vs female; • P<0.05 *cbg*^*+/+*^ vs *cbg*^*-/-*^.

**Fig 6:**
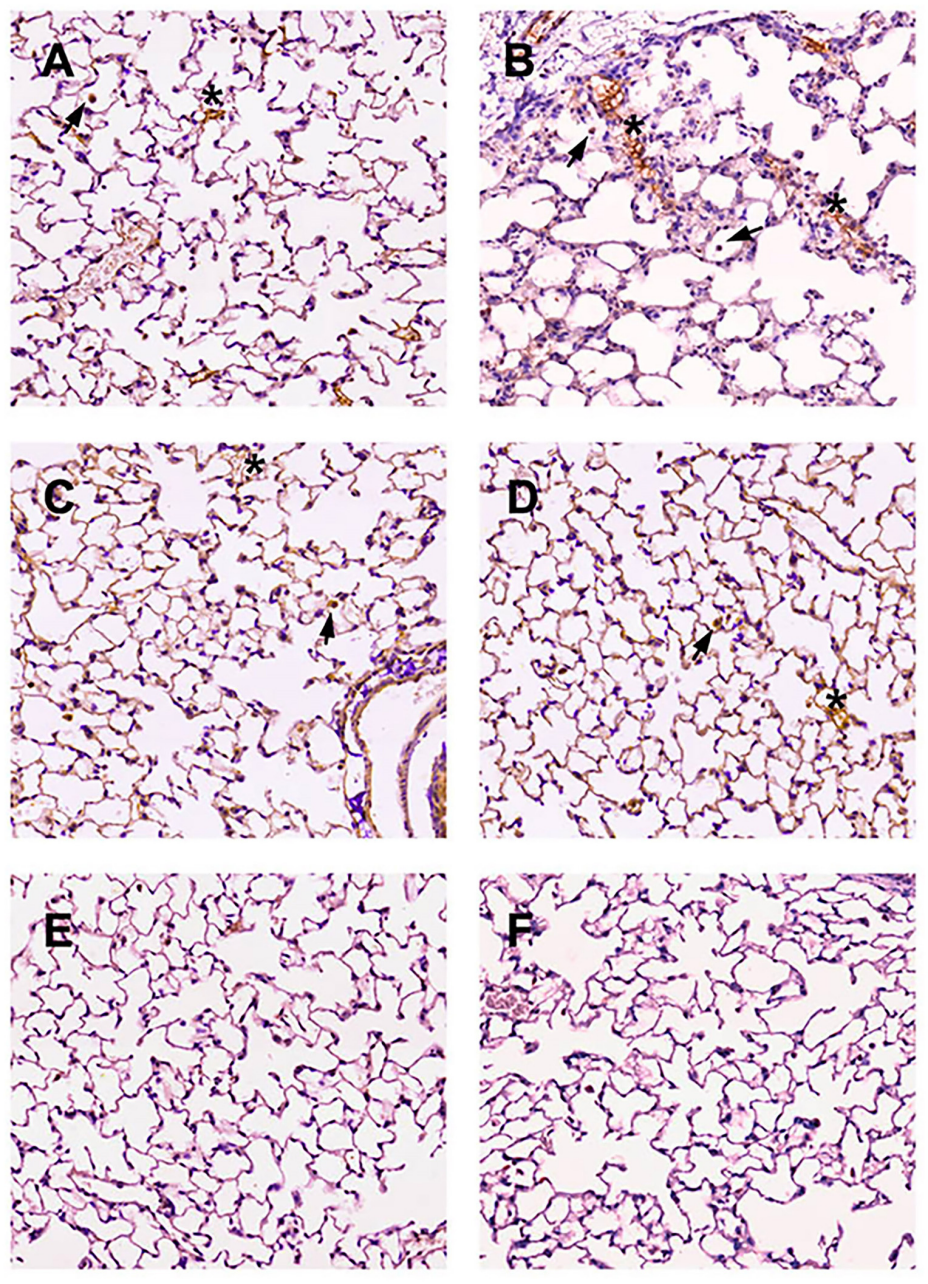
Immunohistochemical analysis of CBG in lung. A) male *cbg*^*+/+*^ control, B) male *cbg*^*+/+*^ pancreatitis, C) female *cbg*^*+/+*^ control, D) female *cbg*^*+/+*^ pancreatitis, E) mice *cbg*^*-/-*^ control and F) negative control without primary antibody. In all *cbg*^*+/+*^ groups there were stained alveolar epithelial cells indicated with an asterisk and some macrophages indicated with an arrow. There was an increase of positive cells in males after induction of pancreatitis. No differences were observed between controls and pancreatitis in females.

In the lung tissue, CBG expression in males *cbg*^*+/+*^ was increased after induction of pancreatitis. By contrast, CBG expression in females *cbg*^*+/+*^ after induction of acute pancreatitis remained unmodified (Fig 5A) resulting in lower levels than males *cbg*^*+/+*^ with pancreatitis. As occurred in liver, residual expression of CBG was observed in the lung of *cbg*^*-/-*^ animals. Similar pattern was found when analyzing by western blot the levels of CBG in tissue (Fig 5B). Finally, immunohistochemical analysis confirmed the induction of CBG in lung from male mice and the lack of induction in female (Fig 6). CBG expression was observed in the alveolar epithelial cells and macrophages. Although there was a residual RNA expression of CBG in *cbg*^*-/-*^ mice, no staining was observed in the corresponding histological samples.

**Fig 7:**
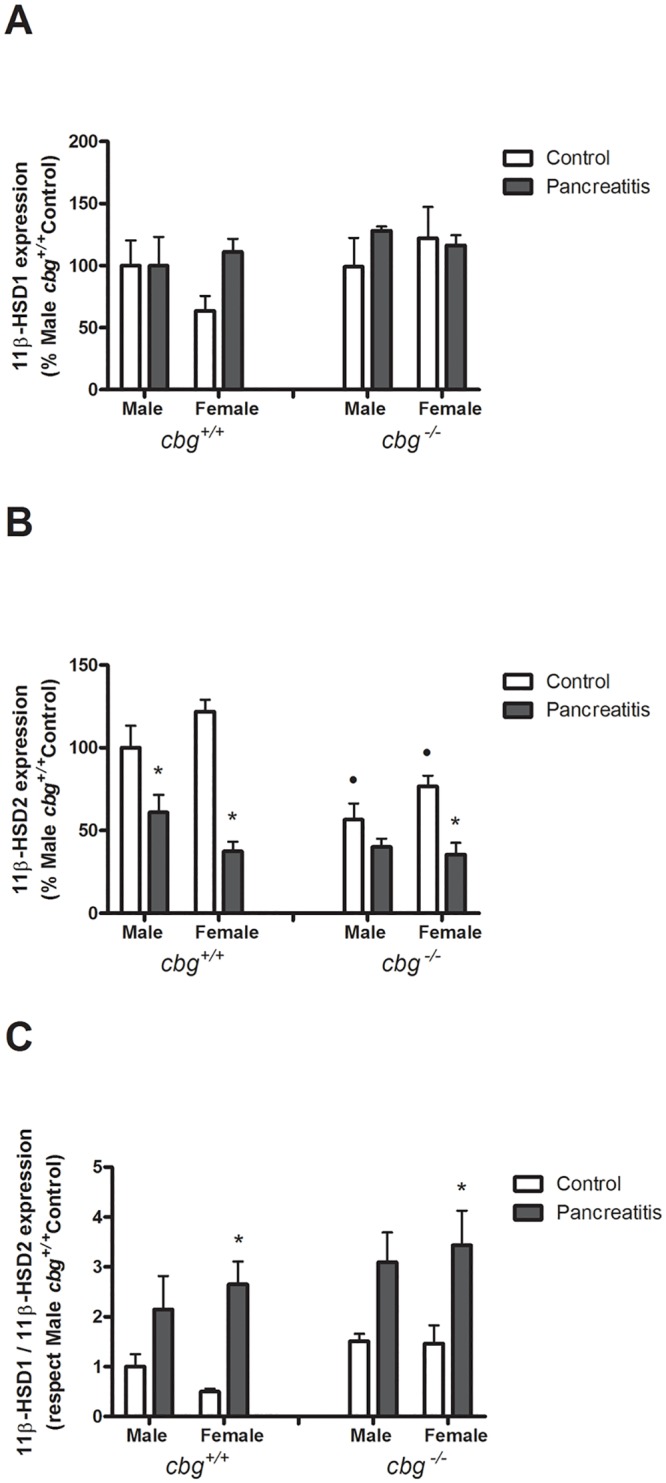
(A) Lung expression of 11β-HSD1, (B) of 11β-HSD2 and (C) the ratio 11β-HSD1/11β-HSD2 (C). The expression of 11β-HSD1 only showed a not significant increase induced by pancreatitis in *cbg*^*+/+*^ female. In contrast, pancreatitis decreased in both genders the expression of 11β-HSD2. *Cbg*^*-/-*^ mice showed lower expression of 11β-HSD2. The ratio 11β-HSD1/11β-HSD2 was increased by pancreatitis in all groups studied but only significantly in females for both genotypes. Two Way Anova: Lung expression of 11β-HSD1: Treatment P = 0.2238, Sex P = 3996, Interaction P = 5188; Lung expression of 11β-HSD2: Treatment P<0.0001, Sex P = 0.0012, Interaction P = 0.0057; ratio11β-HSD1/11β-HSD2: Treatment P<0.0001, Sex P = 0.1818, Interaction P = 0.7634; Bonferroni Post test: * P<0.05 control vs pancreatitis; + P<0.05 male vs female; • P<0.05 *cbg*^*+/+*^ vs *cbg*^*-/-*^.

In order to evaluate the capability of corticosterone synthesis or degradation in lung, we determined the expression of 11β-hydroxysteroid dehydrogenase type 1 (11β-HSD1) and type 2 (11β-HSD2). No differences were observed in 11β-HSD1 expression in any condition, nor due to genotype, cerulein treatment or gender (Fig 7A).

With respect to 11β-HSD2 expression (Fig 7B), gender differences were not found, nor in *cbg*^*+/+*^ nor in *cbg*^*-/-*^ mice. Pancreatitis, in males and females *cbg*^*+/+*^ mice, significantly reduced the expression of 11β-HSD2. In the *cbg*^*-/-*^ mice, control expression was lower than in *cbg*^*+/+*^ and pancreatitis also resulted in a decreased expression although in this case only in females achieved significantly values.

Since 11β-HSD1 catalyzes the conversion of 11-dehydrocorticosterone to the active corticosterone, whereas 11β-HSD2 catalyzes the opposite reaction, the ratio 11β-HSD1/11β-HSD2 indicates the changes in the local glucocorticoid metabolism (Fig 7C). Induction of pancreatitis increases the ratio 11β-HSD1/11β-HSD2 in both genders, indicating a higher level of local corticosterone activation. Similar effect was observed in the *cbg*^*-/-*^ mice.

## Discussion

The progression of the inflammatory response results from the balance of several pro- and anti-inflammatory mediators. Cytokines, free radicals, lipid mediators and activated enzymes promote the activation of inflammatory pathways, not only locally, but also in circulating cells and in distant organs [19]. These effects are counteracted by the release of anti-inflammatory cytokines as IL-10 [20] or proteins as pancreatitis associated protein [21]. However, glucocorticoids are known to play a major role in the control of the inflammatory response and corticosteroid insufficiency has been reported in patients with inflammatory diseases as acute pancreatitis [5][22].

It is known that the glucocorticoids ability to regulate the inflammatory response is strongly related with their availability on the site of inflammation. Tissue availability of glucocorticoids, namely the proportion of glucocorticoid able to bind to the receptor and carry out a response, depends of its synthesis at adrenal gland [23], its binding to the CBG [24], as well as its activation or inhibition catalyzed locally by the intracellular activity of enzymes 11β-HSD1 and 11β-HSD2 [25]. Elastase from activated neutrophils can cleave CBG and promote the release of bound glucocorticoids at the inflammation precise site [26]. An active role of CBG in glucocorticoid response is supported by observations in CBG deficient mice, that show a higher mortality in response to septic shock with lung particularly affected [15]. In acute pancreatitis the respiratory distress syndrome is the most relevant problem associated [1]. Therefore, we aimed to study the changes in blood and specifically lung glucocorticoid availability to cope with inflammation and the lung injury associated in CBG deficient mice with acute pancreatitis. Also we included males and females since the known sexual dimorphism of CBG could be an important modulator that would lead to different responses in each gender.

In general the induction of pancreatitis in mice resulted in higher levels of circulating lipase that were more pronounced in female *cbg*^*+/+*^ animals. This difference was not observed in *cbg*^*-/-*^ mice and the levels achieved were similar to that observed in male *cbg*^*+/+*^ mice (Fig 1A). In pancreas and lung the induction of pancreatitis also increased the inflammatory response without differences between genders or *cbg*^*+/+*^ mice versus the *cbg*^*-/-*^ (Fig 1B and 1C). The pancreatitis severity score determined by histological study (Fig 2) showed pancreatic acinar necrosis in female but not in male while a minor lung alveolar thickening was observed in female. Differences triggered by the genotype were only observed in the females with a slightly more damaged pancreas in *cbg*^*-/-*^ compared to *cgb*^*+/+*^ mice. The results clearly state that pancreatitis was induced but failed to show significant differences in the severity of the disease as a consequence of circulating CBG levels. Whereas serum lipase suggested more severity in females *cbg*^*+/+*^ other parameters showed no differences between *cbg*^*+/+*^ vs *cbg*^*-/-*^ as pancreas and lung MPO activity or severity scores in the case of males. Finally, the histological analysis in females suggested more severity in cbg^-/-^ mice. To clearly determine if there are differences between *cbg*^*+/+*^ and *cbg*^*-/-*^ mice in the severity of the pancreatitis induced by cerulein further studies are needed.

On the other hand, when analyzing the total serum corticosterone levels in the *cbg*^*+/+*^ mice (Fig 3A) we observed that there was a clear difference between male and female, showing females the expected higher levels than males [27], however no changes due to pancreatitis. By contrast, *cbg*^*-/- *^ control mice showed low levels of corticosterone without differences between male and female. Interestingly, pancreatitis significantly increased corticosterone in *cbg*^*-/-*^ animals in a similar magnitude in both genders, until the levels of cbg^*+/+*^ mice (Fig 3A). We were very careful inducing pancreatitis and obtaining samples at the same time of day in all animals to avoid errors due to circadian changes in circulating corticosterone, a factor that may cause confusion as it has been demonstrated by Richard et al. [28].

The low levels observed in circulating corticosterone in *cbg*^*-/-*^ mice agree with those reported by Petersen et al [15]. It has been suggested that this carrier protein have specific roles in the regulation of circulating turnover, local delivery, and cellular signal transduction of steroid hormones [29]. The total corticosterone levels are regulated through hypothalamus-pituitary-adrenal (HPA) axis by a negative feed-back mechanism. It is accepted that the biologically active corticosterone is the free fraction circulating in blood while the corticosterone bound to CBG is unable to enter the cell and, therefore, is considered inactive. In our study, *cbg*^*-/-*^ mice display increased free levels, i.e. active glucocorticoid. Despite that, it has been described that *cbg*^*-/-*^ mice exhibit higher levels of ACTH, a sign of HPA activity [15]. Further, we have observed that the *cbg*^*-/-*^ mice show an adrenal gland weight significantly larger than *cbg*^*+/+*^ mice (unpublished results) indicating a major activity of the gland. These features suggest that the HPA axis is irresponsive to the negative feed-back exerted by free corticosterone in *cbg*^*-/-*^ pointing to a role of CBG in the HPA axis control and regulating corticosterone synthesis.

In addition to the higher levels of total serum corticosterone in female (Fig 3A) there were also clear differences in circulating CBG (Fig 4A), and CBG binding capacity (Fig 3B) when comparing males and females, as expected, having the females higher values in all these parameters [27]. It is noteworthy that in *cbg*^-/-^ mice, the gender difference in corticosterone levels was not observed in controls neither in pancreatitis induced animals. The role of CBG in the gender differences on circulating glucocorticoids have been observed recently in a model of emotional stress [30] where was found that the sex differences normally described for stress reactivity disappear when estrogens are removed in ovariectomiced mice. Furthermore, the *cbg*^*-/-*^ mice showed no differences between males and females in stress reactivity and neither in the absence of estrogens. Nevertheless, there are some differences in the response of *cbg*^*-/-*^ animals to stress or to acute pancreatitis inflammation. While under the stress, *cbg*^*-/-*^ animals failed to increase corticosterone levels [30], in our model they show the ability to increase its levels in response of inflammation. Therefore, CBG seems determinant to maintain the levels of circulating' glucocorticoids and their gender differences.

It is well known that CBG regulates the amount of free corticosterone released during inflammation [26] but our results indicate that this protein also plays a role in modulating the concentration of total circulating corticosterone. The highest levels of serum CBG detected in females probably induces a high buffering effect thus promoting the synthesis of more total corticosterone in order to achieve the concentrations of free hormone necessary for homeostasis. Although liver is the main site of synthesis for CBG which is secreted into the blood to transport corticosterone, some reports described the generation of this globulin in other organs as kidney, pancreas, placenta, hypothalamus and adipose tissue [9] [31] [10] [32][11] where its function remains unknown. The cDNA of CBG was cloned firstly from a cDNA library of liver and lung [33] and a CBG precursor has been described in pleural effusions from lung adenocarcinoma patients [34], but despite this, the significance of its presence in the lung has been little explored. Here, we evaluated the presence of CBG in the lung. RNA expression, western blot and immunohistochemistry analysis revealed that lung also generate CBG. Interestingly, the local expression of CBG in the lung (Fig 5) showed a completely opposite pattern than that observed in liver (Fig 4B), having males higher expression of CBG than females. This fact suggests a different role for CBG in these organs. While liver is the main source for circulating CBG, the role in the lung appears to be restricted to the control of local corticoids bioavailability.

Although the circulating levels of corticosterone reflect the general systemic anti-inflammatory response, the action of corticosterone also depends on the local activity of the enzymes 11β-HSD1 and 11β-HSD2. In general, it is accepted that in response to inflammation the levels of 11β-HSD1 are increased in order to potentiate the anti-inflammatory effects of glucocorticoids. Less information is available on 11β-HSD2. In vitro it is known that pro-inflammatory cytokines, as TNFα, upregulate 11β-HSD1 [35] and downregulate 11β-HSD2 [36]. Downregulation of 11β-HSD2 without changes in 11β-HSD1 has also been described in skin lesions of leprosy patients [37]. In our study, the main change observed in the lung was the decreased levels of the inactivating enzyme 11β-HSD2 elicited by pancreatitis without modification of 11β-HSD1 expression. Irrespective of gender or genotype, pancreatitis resulted in similar high levels of 11β-HSD1/11β-HSD2 ratio, evidencing the expected corticosterone activation status triggered by an inflammatory stimulus. Altogether suggests that in lung the anti-inflammatory actions of glucocorticoids would be determined by 11β-HSD2.

Interestingly, in control *cbg*^*-/-*^ mice lung expression of 11β-HSD2 showed lower levels than *cbg*^*+/+*^. This fact could be a mechanism to increase corticosterone levels into the tissue to counteract the impaired availability of tissue corticosterone observed in *cbg*^*-/-*^ [15].

In conclusion, our results indicate that the role of CBG is not only restricted to act as a carrier of corticosteroids and a modulator of its tissue availability. It is also an important factor involved in the gender differences observed in corticosteroids levels. CBG is generated in the lung showing a pattern of expression opposite to the liver either in the response to the acute pancreatitis as in the sexual dimorphism. These differences between liver and lung suggest that CBG has a specific, and yet unexplored, role in the lung.
